# Choroidal and retinal thickness in patients with vitamin C deficiency using swept-source optical coherence tomography

**DOI:** 10.1186/s12886-022-02530-8

**Published:** 2022-07-18

**Authors:** Yiwen Qian, Luoziyi Wang, Xinfang Qiang, Huan Weng, Jing Jiang, Xin Che, Qingjian Li, Zhiliang Wang

**Affiliations:** 1 Department of Ophthalmology, Huashan Hospital of Fudan University, No. 12 Middle Urumqi Road, Shanghai, 200040 China; 2 Department of Ophthalmology, Xiang’an Hospital of Xiamen University; Fujian Provincial Key Laboratory of Ophthalmology and Visual Science; Eye Institute of Xiamen University; School of Medicine, Xiamen University, Xiamen, Fujian China

**Keywords:** Vitamin C, Choroidal thickness, Retinal thickness, SS-OCT, Reactive oxygen species

## Abstract

**Background:**

To investigate the effects of vitamin C on central retinal thickness and choroidal thickness.

**Methods:**

A total of 69 patients diagnosed with vitamin C deficiency and 1:1 age- and gender-matched 69 healthy individuals with normal serum vitamin C were included in this study. Demographic characteristics of the individuals were collected. All patients underwent a comprehensive ophthalmic examination. Subfoveal choroidal thickness and retinal thickness were measured using a swept-source optical coherence tomography (SS-OCT).

**Results:**

The average retinal thickness was 269.07 ± 13.51 μm in the vitamin C deficiency group and 276.92 ± 13.51 μm in the control group. The average choroidal thickness was 195.62 ± 66.40 μm in the in the vitamin C deficiency group and 238.86 ± 55.08 μm in the control group. There was a significant decrease in both average choroidal thickness and retinal thickness in vitamin C deficiency group compared with normal individuals (*p* < 0.001, and = 0.001 respectively).

**Conclusion:**

The central retinal and choroidal thickness were thinner in vitamin C deficiency group compared with normal individuals. These findings suggested that vitamin C deficiency might play an important role in retinal and choroidal diseases.

## Background

The retina, a layer of nervous tissue, is rich in polyunsaturated fatty acids (PUFA) and highly susceptible to lipid peroxidation [1]. The prolonged exposure to radiant energy, elevated metabolic activity and oxygen consumption may lead to reactive oxygen species (ROS)-mediated apoptosis in retina cells, especially the photoreceptor cells and retinal pigment epithelium [2]. Prior studies have observed oxidative stress is relevant to the pathophysiology of retinal diseases, such as age-related macular degeneration (AMD), diabetic retinopathy and glaucoma [2–4].

The choroid, with the highest blood flow rates in the body, is of vital importance to the etiology and physiology of retina by providing oxygen and nutrients to the outer retinal layers and removing waste products from the eye. Choroid plays a pivotal role in many retinal diseases, such as age-related macular degeneration (AMD), glaucoma, uveitis, diabetic retinopathy, etc. [5, 6]. Accurate measurement of choroidal thickness is an important step in monitoring disease onset and progression that lead to choroidal thinning.

For the relationship between ROS and various retinal diseases, mechanism of removal of free radicals by antioxidant has been studied in recent years [7, 8]. Vitamin C is an antioxidant for humans, with pleiotropic functions such as antioxidation and a cofactor of a large number of biosynthetic and gene regulatory enzymes. Supplementation of vitamin C also contributes to immune defense by improving the function of the human innate and adaptive immune system, such as antimicrobial and natural killer cell activities, lymphocyte proliferation [9]. A cumulative number of studies on animal and human with dietary supplementation of antioxidants (including vitamin C) were found to restore glutathione level and inhibit oxidative damage in the retina [10].

The retina and choroid both possess a high concentration of vitamin C transporters (sodium-vitamin C cotransporter) SVCT2 [11]. Therefore, it is important to investigate the effect of antioxidant of vitamin C for the oxidative stress induced damage in retina and choroid. The novel swept-source optical coherence tomography imaging (SS-OCT), with a longer central wavelength (1050 nm), has the advantage of deeper penetration through the retinal pigment epithelium (RPE) [12]. It enables noninvasive visualization and measurement of the choriocapillaris and choroidal vasculature, opening up a new world of research in this previously underexplored ocular tissue.

To the best of our knowledge, there have been no studies evaluating macular retinal and choroidal thickness with vitamin C deficiency. The aim of this study is to evaluate the relationship of the vitamin C level with retinal and choroidal vasculature in normal individuals. Thus, it may help in understanding the etiology of oxidative stress on retina and choroid.

## Methods

The retrospective, cross-sectional study included a total of 2185 participants who referred for medical checks including vitamin analysis performed at Huashan Hospital, Fudan University, Shanghai, China, from May 2019 to December 2019, conforming to the tenets of the Declaration of Helsinki. Ethical approval (KY2016–274) was obtained from the Institutional Review Board of Huashan Hospital affiliated with Fudan University. All subjects enrolled in the study provided written informed consent before undergoing the examinations. Blood samples of various vitamins of the participants were taken on the same morning for analysis. Vitamin C deficiency is defined as vitamin C below standard value (34 μmol/L—114 μmol/L). The participants were grouped into vitamin C deficiency (< 34 μmol/L) and normal group (≥34 μmol/L). Other vitamins of all 138 participants were within normal range: vitamin B2 (> 200 μg/L), vitamin B6 (14.60–72.9 μmol/L), vitamin B12 (200-900 pg/mL) and vitamin E (10.0–15.0 μg/L), All participants underwent comprehensive ocular examination including autorefractometer, and slit-lamp biomicroscopy. The study participants had best corrected visual acuities (BCVA) of 20/25 or more, a refractive error in the range + 3.0 to − 3.0 diopters and intraocular pressure (IOP) between 10 mmHg and 21 mmHg. Those with systemic diseases such as hypertension, diabetic mellitus, cardiovascular disease and renal impairment and retinal diseases such as inherited retinal dystrophies, uveitis and AMD or a history of ophthalmic surgery that may have affected the choroidal vascular network were excluded. In addition, poor OCT image due to media opacities or unstable fixation were also excluded. 69 eligible subjects with vitamin C deficiency were enrolled in and 1:1 age- and sex-paired normal vitamin C subjects were included for control group.

### Swept-source optical coherence tomography imaging

OCT images were obtained with a SS-OCT (DRI OCT-1 Atlantis, Version 9.31, Topcon Co., Tokyo, Japan) which overcomes the scattering of light on the choroid due to longer wavelength of approximate 1050 nm**.** The scan speed in the SS-OCT device is 100,000 A-scans per second, providing more accurate images of the retina and choroid. Twelve equidistant radial line scans with a length of 9 mm were performed centered on the fovea of the macula. The macular retinal thickness and choroidal thickness were defined respectively as the distance from the internal limiting membrane (ILM) to the basal edge of the RPE and the distance from the outer border of the RPE to the chorioscleral interface (CSI). Thickness map were created automatically in accordance with the standard Early Treatment Diabetic Retinopathy Study (ETDRS) subfield. The average thicknesses of the nine sectors were measured automatically according the standard ETDRS grid. For every OCT scans, each segmented layer line can be manually adjusted to avoid automatic measurement errors. Only the right eye of each participant was assessed for statistical analysis. The OCT scanning were performed during the same daily interval (8-10 am) to avoid diurnal variations in retinal and choroidal thickness and performed by the same skilled technician who was blinded to the blood results.

### Statistical analysis

SPSS statistical analysis software (SPSS, Version 24.0, IBM Inc., Chicago, IL, USA) was used for all statistical analyses. Values were expressed as mean ± standard deviation (SD). Continuous data were compared using the Student’s t-test. Pearson’s correlation coefficient was used to test the relationships between choroidal thickness, retinal thickness and vitamin C. Statistical significance was defined as 2-tailed *p*-value < 0.05.

## Results

Table 1 listed the baseline characteristics of the participants. Sixty nine patients with vitamin C deficiency and 69 healthy controls were matched by sex and age (1:1 pair-wise matching). The mean vitamin C level was 31.25 ± 1.78 (range from 25.09 to 33.67) μmol/L in vitamin C deficiency group and 39.53 ± 4.58 (range from 34.09 to 58.53) μmol/L in control group. The average age of the vitamin C deficiency group was 57.26 ± 14.16 years old in vitamin deficiency group and 58.13 ± 12.37 years old in control group. No significant differences in blood pressure and refractive error were noted between the vitamin C deficiency group and control group. Other vitamins including vitamin E and vitamin B2, B6, B12 were all within normal range and showed no significant differences between the two groups..

**Table 1:** Demographic characteristics

Parameter	Vitamin C deficiency group	Control group	*P* Value
Patient, n	69	69	–
Eye, n	69	69	–
Gender, n			
Male, n	37	37	
Female, n	32	32	
Age, years	57.26 ± 14.16	58.13 ± 12.37	0.699^a^
Range, years	30–75	30–75	
SBP, mmHg	127.49	124.46	0.193^a^
DBP, mmHg	76.81	75.35	0.352^a^
Refractive error, D	−0.875	−0.596	0.260^a^
Vitamins			
Vitamin E	11.79	11.85	0.749^a^
Vitamin B2	248.43	246.80	0.806^a^
Vitamin B6	20.02	20.36	0.700^a^
Vitamin B12	243.68	236.10	0.194^a^

^a^T-test. *SBP* systolic blood pressure, *DBP* diastolic blood pressure

**Fig. 1:**
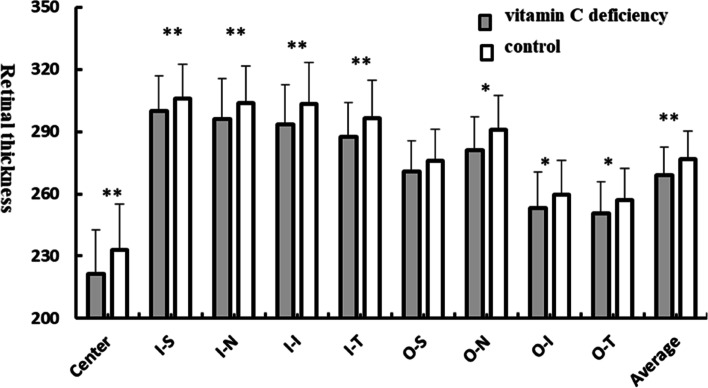
The retinal thickness of nine sectors of the ETDRS grid in the vitamin C deficiency group compared with the controls. ^*^ *p* < 0.05, ^**^ *p* < 0.01. I-S, innr superior; I-N, inner nasal; I-I, inner inferior; I-T, inner temporal; O-S, outer superior; O-N, outer nasal; O-I, outer inferior; O-T, outer temporal

Data showed the average retinal thickness was significantly decreased in the vitamin C deficiency group compared with the control group (269.07 ± 13.51 μm vs 276.92 ± 13.51 μm, *p* = 0.001). What’s more, the retinal thickness in the vitamin C deficiency group was thinner at all nine sectors of retina compared with the control group, among which the center and the inner sectors of the retina have the most significant decrease of retinal thickness (Table 2 and Fig. 1).

**Table 2:** Retinal thickness of nine sectors of the ETDRS grid

Retinal thickness	Vitamin C deficiency group *n* = 69	control group *n* = 69	*P* Value
Center, μm	221.45 ± 21.31	232.95 ± 22.10	0. 002^a^
Inner superior, μm	299.98 ± 16.92	306.16 ± 16.45	0. 031^a^
Inner nasal, μm	296.25 ± 19.53	303.89 ± 17.71	0. 017^a^
Inner inferior, μm	293.35 ± 19.16	303.48 ± 19.71	0. 003^a^
Inner temporal, μm	287.53 ± 16.32	296.56 ± 18.39	0. 002^a^
Outer superior, μm	270.97 ± 14.65	275.83 ± 15.46	0. 060^a^
Outer nasal, μm	281.17 ± 15.97	290.96 ± 16.69	0. 001^a^
Outer inferior, μm	253.33 ± 17.23	259.81 ± 16.15	0. 024^a^
Outer temporal, μm	250.69 ± 14.98	256.83 ± 15.41	0. 012^a^
Average thickness, μm	269.07 ± 13.51	276.92 ± 13.51	0. 001^a^

^a^T-test

**Fig. 2:**
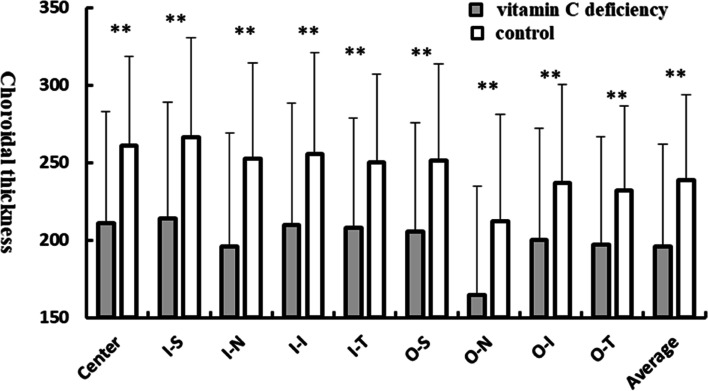
The choroidal thickness of nine sectors of the ETDRS grid in the vitamin C deficiency group compared with the controls. ^*^ *p* < 0.05, ^**^ *p* < 0.01. I-S, innr superior; I-N, inner nasal; I-I, inner inferior; I-T, inner temporal; O-S, outer superior; O-N, outer nasal; O-I, outer inferior; O-T, outer temporal

Data showed that average choroidal thickness was significantly thinner in the vitamin C deficiency group than in the control group (195.06 ± 66.35 μm vs 238.53 ± 56.20 μm, *p* < 0.001). In addition, the choroidal thickness of all nine ETDRS sectors showed a significant decrease in the vitamin C group compared with the control group (Table 3 and Fig. 2).

**Table 3:** Choroidal thickness of nine sectors of the ETDRS grid

Choroidal thickness	Vitamin deficiency group *n* = 69	control group *n* = 69	*P* Value
Center, μm	210.93 ± 71.66	260.87 ± 57.82	< 0. 001^a^
Inner superior, μm	214.06 ± 74.91	266.52 ± 63.79	< 0. 001^a^
Inner nasal, μm	196.07 ± 72.94	252.31 ± 62.00	< 0. 001^a^
Inner inferior, μm	209.94 ± 78.65	255.73 ± 65.37	< 0. 001^a^
Inner temporal, μm	207.80 ± 70.85	250.04 ± 56.73	< 0. 001^a^
Outer superior, μm	205.46 ± 70.34	251.04 ± 62.37	< 0. 001^a^
Outer nasal, μm	164.22 ± 70.61	212.32 ± 68.87	0. 002^a^
Outer inferior, μm	200.20 ± 72.09	236.59 ± 63.48	0. 001^a^
Outer temporal, μm	197.08 ± 69.46	232.07 ± 54.61	0. 001^a^
Average thickness, μm	195.62 ± 66.40	238.86 ± 55.08	< 0. 001^a^

^a^T-test

The correlations of retinal thicknesses and Choroidal thicknesses with blood vitamin C level were shown in Tables 4 and 5 separately. Both average retinal thicknesses and choroidal thicknesses had positive correlations with vitamin C in the entire population (*r* = 0.246, *p* = 0.004 for retinal thickness and *r* = 0.295, *p* < 0.001 for choroidal thickness).

**Table 4:** Correlation analysis results between retinal thickness and Vitamin C

Retinal thickness		Center	I-S	I-N	I-I	I-T	O-S	O-N	O-I	O-T	Average
VC	r	0.258^**^	0.232^**^	0.223^**^	0.294^**^	0.261^**^	0.130	0.198^*^	0.166	0.182^*^	0.246^**^
	p	0.002	0.006	0.009	< 0.001	0.002	0.128	0.017	0.052	0.033	0.004

^*^ *p* < 0.05, ^**^ *p* < 0.01

*I-S* innr superior, *I-N* inner nasal, *I-I* inner inferior, *I-T* inner temporal, *O-S* outer superior, *O-N* outer nasal, *O-I* outer inferior, *O-T* outer temporal, *VC* vitamin C

**Table 5:** Correlation analysis results between choroidal thickness and Vitamin C

Choroidal thickness		Center	I-S	I-N	I-I	I-T	O-S	O-N	O-I	O-T	Average
V C	r	0.301^**^	0.301^**^	0.306^**^	0.265^**^	0.295^**^	0.269^**^	0.289^**^	0.224^*^	0.273^**^	0.295^**^
	p	< 0.001	< 0.001	< 0.001	0.002	< 0.001	0.001	< 0.001	0.09	0.001	< 0.001

^*^ *p* < 0.05, ^**^ *p* < 0.01

*I-S* innr superior, *I-N* inner nasal, *I-I* inner inferior, *I-T* inner temporal, *O-S* outer superior, *O-N* outer nasal, *O-I* outer inferior, *O-T* outer temporal, *VC* vitamin C

The differences between men and women with vitamin C deficiency, both for the retinal and for the choroidal thickness were demonstrated in Tables 6 and 7. In our current data (*n* = 69), men (*n* = 36) had thicker chorodial and retinal thicknesses than women (*n* = 33) in Vitamin C deficiency individuals. While no significant differences were noted in the average retinal and choroidal thicknesses between men and women (*p* = 0.427 and *p* = 0.557 separately). Retinal thickness in the center, inner superior, inner nasal and inner temporal sectors showed significant differences between men and women in Vitamin C deficiency subjects (Table 6). While all nine sectors of choroidal thickness showed no significant differences in men and women in Vitamin C deficiency subjects (Table 7).

**Table 6:** Retinal thickness in Vitamin C deficiency individuals

Retinal thickness	Men *n* = 36	Women *n* = 33	*P* Value
Center, μm	228.22 ± 23.58	214.07 ± 15.78	0. 005^a^
Inner superior, μm	305.01 ± 15.43	294.50 ± 16.99	0. 009^a^
Inner nasal, μm	303.33 ± 17.64	288.54 ± 18.76	0. 001^a^
Inner inferior, μm	296.69 ± 20.58	289.71 ± 17.13	0. 130^a^
Inner temporal, μm	293.12 ± 15.23	280.99 ± 15.27	0. 002^a^
Outer superior, μm	270.50 ± 15.86	271.48 ± 13.45	0.783^a^
Outer nasal, μm	280.46 ± 17.85	281.94 ± 13.86	0. 702^a^
Outer inferior, μm	249.66 ± 18.02	257.33 ± 15.62	0. 064^a^
Outer temporal, μm	251.70 ± 15.30	249.18 ± 14.80	0. 488^a^
Average thickness, μm	270.31 ± 13.38	267.70 ± 13.73	0. 427^a^

^a^T-test

**Table 7:** Choroidal thickness in Vitamin C deficiency individuals

Choroidal thickness	Men *n* = 36	Women *n* = 33	*P* Value
Center, μm	213.44 ± 75.96	208.19 ± 67.73	0.762^a^
Inner superior, μm	220.35 ± 82.30	207.19 ± 66.50	0.466^a^
Inner nasal, μm	193.70 ± 75.41	198.67 ± 71.23	0.779^a^
Inner inferior, μm	214.81 ± 85.86	204.62 ± 70.89	0.592^a^
Inner temporal, μm	208.50 ± 74.54	207.03 ± 67.73	0.932^a^
Outer superior, μm	214.25 ± 78.14	195.87 ± 60.46	0.276^a^
Outer nasal, μm	164.50 ± 77.87	163.92 ± 62.95	0.973^a^
Outer inferior, μm	208.13 ± 77.16	191.54 ± 66.21	0.340^a^
Outer temporal, μm	201.03 ± 74.77	192.76 ± 64.05	0.623^a^
Average thickness, μm	200.16 ± 72.30	190.67 ± 60.01	0.554^a^

^a^T-test

## Discussion

Oxidative stress due to vitamin C deficiency has been implicated as critical pathogenic factors contributing to the etiology of retinal and choroidal diseases [10]. A cumulative research has focused on the protection of retinal and choroidal physiology and function by vitamin C supplementation due to its antioxidative properties. In our current study, we found that both the retinal and choroidal thickness significantly decreased in the vitamin C deficiency group compared with age- and gender- matched normal vitamin C individuals. This result might suggest a close correlation between vitamin C deficiency with the retinal and choroidal diseases.

An imbalance between ROS and antioxidants in the body may result in excessive generation of ROS, including hydrogen peroxide, superoxide and peroxynitrate. These insults may damage cellular macromolecules and organelles and promote cell death via apoptosis. Vitamin C is an important antioxidant which can prevent the oxidative damage to the retina [13]. A high level of vitamin C in retina alleviates free radicals and improves immune cell function generated by its high metabolic activity [9]. As a result, there has been persistent interest in antioxidant approaches (such as vitamin C) to combat oxidative stress in retinal diseases.

The retina and choroid possess a high-affinity transport system for vitamin C and the highest concentration of vitamins was located in the RPE followed by the outer segments of the photoreceptor cells [14]. Both in vivo and in vitro experiments have verified the prominent effect of vitamin C in retina health [14]. In animal model of vitamin C deficiency, short-term low vitamin C level would induce oxidative stress in the retinas of young guinea pigs [15, 16]. In a model of porcine hypercholesterolemia, vitamins C improved retinal structure alteration in transmission electron microscopy by preventing oxidative stress and nitric oxide metabolites [17]. In an oxidative stress model induced by hydrogen peroxide and ultraviolet B irradiation of ARPE-19 cells, antioxidative effect of vitamin C could result in increment in cell viability and reduction in intracellular ROS level [18]. The study of AREDS showed that treatment of high dose antioxidant supplements in patients with intermediate AMD was effective in retarding the progression of AMD compared with placebo [19, 20].

Mechanisms by which vitamin C deficiency correlated with retinal and choroidal thickness are multifactorial. Vitamin C deficiency may increase oxidative stress which directly contributes to apoptosis, damaging of retina cells (especially the photoreceptor cell) and activation of retinal immune system. The decrease of retinal thickness may due to the thinning of the photoreceptor cell layer caused by apoptosis, shortening of the photoreceptor cell outer segment. In an animal study, spaceflight inducing oxidative damage resulted an increase in photoreceptor cone damage, and reduction of thickness of the retinal outer nuclear layer (ONL), retinal inner nuclear layer (INL), RPE, and choroid layers of the eye in C57BL/6 mice [21]. In retinal glial cells, oxidative stress increased the expression of major histocompatibility complex (MHC) II, consequently improving the ability to stimulate T cell proliferation and secretion of cytokines (TNFa) that can induce glial cell apoptosis [22]. As the cones contain more mitochondria and have a higher energy demand than rods, it is much more prone to be affected by oxidative stress [23]. In our study, the center and the inner sectors of the retina showed the most significant difference between the two groups, while the outer sectors had a trend of retinal thinning without significant difference.

The second possibility was role of vitamin C in the function of vascular endothelium [24]. Ascorbate has long been known to enhance endothelial synthesis and deposition of Type IV collagen to form the basement membrane of blood vessels. Cell experiment demonstrated that vitamin C could tighten the endothelial permeability barrier and control endothelial cell proliferation and apoptosis resulting from many dioxygenase involved in endothelial function, proliferation, and survival [24]. Recent studies have found that low vitamin C concentration is linked with vascular disease, such as atherosclerosis and cardiovascular diseases due to endothelial dysfunction [25, 26]. High dose of vitamin C administration showed beneficial function in endothelial function in various artery diseases [27, 28].

Moreover, vitamin C is closely associated with inflammatory regulation. A cumulative researches have confirmed that inflammation is a critical factor contributing to the pathogenesis of many microvascular disorders [29, 30]. Retinal and choroidal thickness showed close relation with inflammatory diseases [31]. Balmforth et al. revealed a choroidal thinning with increased IL-6, TNF-α and endothelin-1 due to inflammation and endothelial dysfunction [32]. These might be the reasons contributing to the significant difference in retinal and choroidal thickness between the two groups.

Although the retina is extremely sensitive to oxidative stress, retinal thickness in the vitamin C deficiency does not have such a remarkable thinning as the choroidal thickness. The possible explanation is the high blood flow of choroidal vasculature compared with retina vessels. Moreover, regulation of blood flow to the retina and the choroid is quite different: retinal flow vasculature can accommodate autogenously, while choroidal flow relies on autonomic regulation.

The present study has several limitations. First, although we have included 138 individuals, we still need a larger population to evaluate the effect of vitamin C in choroidal and retinal vasculature. Secondly, we did not assess the smoking population among the subjects while cigarette was believed to be partially responsible for choroid thinning. Moreover, the duration of vitamin C deficiency of each subject was unknown which might also affect retinal and choroidal thickness. Future prospective studies of serum antioxidants and incident of retina and choroid thinning may help to further clarify the effect of antioxidants on the health of retina and choroid.

## Conclusions

In conclusion, there is a strong relationship of vitamin C deficiency with choroidal and retinal thinning, for the unique antioxidant/protective activities in the retina. Given that patients with vitamin C deficiency might lead to the development choroidal and retinal vasculature problems, it might help new strategies for prevention of treatment for oxidative stress in retinopathy.

## Data Availability

The datasets used and analyzed during the current study are available from the corresponding author upon reasonable request.

## Abbreviations

SS-OCT: swept-source optical coherence tomography
PUFA: polyunsaturated fatty acids
ROS: reactive oxygen species
AMD: age-related macular degeneration
SVCT2: sodium-vitamin C cotransporter
IOP: intraocular pressure
ILM: internal limiting membrane
RPE: retinal pigment epithelium
CSI: chorioscleral interface
ETDRS: Early Treatment Diabetic Retinopathy Study
MHC: major histocompatibility complex
ONL: outer nuclear layer
INL: retinal inner nuclear layer
